# Genetic Landscape of Oral Cavity Squamous Cell Carcinoma

**DOI:** 10.1002/oto2.70194

**Published:** 2026-01-21

**Authors:** Joseph Celidonio, Sree Chinta, John Sebastian de Armas, Dylan Roden

**Affiliations:** ^1^ Otolaryngology–Head and Neck Surgery Rutgers New Jersey Medical School Newark New Jersey USA

**Keywords:** genomic analysis, head and neck, oral cavity, squamous cell carcinoma

## Abstract

**Objective:**

The association, if any, between gene mutations, pathologic features of squamous cell carcinoma of the head and neck, and patient prognosis is unknown. This study investigates the association between common gene mutations in oral cavity squamous cell carcinoma, pathologic features of malignancy, and patient survival.

**Study Design:**

Retrospective database review.

**Setting:**

US hospitals.

**Methods:**

The cBioPortal for Cancer Genomics database was queried for oral cavity squamous cell carcinoma patient data. Statistical analyses were conducted using IBM SPSS v29.

**Results:**

Of the 423 patients included, the majority were male (66.6%), white (89.3%), and current/former smokers (74.1%). Tumor protein p53 (TP53), titin (TTN), and FAT atypical cadherin 1 (FAT1) mutations were present in 72.3%, 31.2%, and 22.0% of patients, respectively. Mutant TP53 was associated with positive extranodal extension compared to wild‐type (WT) TP53 (31.0% vs 18.8%) (*P* = .038) and perineural invasion (59.4% vs 44.3%, *P* = .021). High tumor mutational burden was present in 5.4% of cases. Gene mutations were not associated with differences in median overall survival or disease‐free survival. Multivariable analysis revealed an association between mutant TP53 and the presence of extranodal extension (odds ratio [OR] 2.61, 95% CI 1.05‐6.52, *P* = .039) and perineural invasion (OR 2.14, 95% CI 1.04‐4.42, *P* = .039).

**Conclusion:**

Mutant TP53 was associated with high‐risk pathologic features, including extranodal extension and perineural invasion, but not with inferior survival. A high tumor mutational burden (>10) is rare in oral cavity squamous cell carcinoma. Further research into the interplay between genetic mutations and patient outcomes is needed.

Approximately 300,000 new cases of oral cavity malignancies, 90% of which are squamous cell carcinomas (SCCs), are diagnosed globally each year—these account for 150,000 deaths annually.^1^ The predominant risk factors for oral cavity squamous cell carcinoma (OCSCC) are cigarette smoking, alcohol consumption, and betel nut use.^2^, ^3^ Although the 5‐year overall survival (OS) of early‐stage OCSCC is 90%, the 5‐year OS rate is about 50% for patients who have locally advanced disease.^3^, ^4^, ^5^ Accurate staging of patients with OCSCC is essential for prognostication and for treatment decisions. Currently, tumor mutations are not considered in the American Joint Committee on Cancer (AJCC) staging manual, nor are they considered in the National Comprehensive Cancer Network (NCCN) treatment guidelines for p16‐negative head and neck squamous cell carcinoma (HNSCC). However, the utility of molecular testing and next‐generation sequencing (NGS) in prognostication and treatment is evident in many tumor types. A non‐exhaustive list includes assessing for estrogen receptor, progesterone receptor, and HER2 status in breast cancer, epidermal growth factor receptor mutations and anaplastic lymphoma kinase rearrangements in non‐small cell lung cancer, and B‐rapdily accelerated fibrosarcoma mutational status in anaplastic thyroid cancer.^6^ Additionally, the recent KEYNOTE‐689 trial demonstrated superior survival with the use of immunotherapy in patients with locally advanced, resectable HNSCC with a combined positive score (CPS) of ≥1.^7^ In this study, we sought to investigate the relationships between tumor mutations commonly found in OCSCC with established prognostic factors, such as extranodal extension (ENE) and perineural invasion (PNI).^8^

Tumor protein p53 (TP53) is the most commonly mutated gene in OCSCC, as is true for many other cancer types including ovarian, colorectal, esophageal, and others.^9^, ^10^ TP53 is proposed to drive tumorigenesis by altering the tumor microenvironment to reduce the pro‐inflammatory immune response and increase the proliferation, invasion, and metastasis of tumor cells.^11^ TP53 mutation is thought to confer worse survival in head and neck cancer, and this is the basis for the currently enrolling ECOG‐ACRIN 3132 (EA3132) trial.

We aimed to utilize cBioPortal, an open‐source collection of cancer genomic data sets, to identify the demographics and tumor characteristics associated with common gene mutations. We assessed several gene mutations, which were selected based on which mutations have previously shown prognostic significance in OCSCC (eg, TP53, caspase 8 [Casp8], notch receptor 1 [NOTCH1], and cyclin‐dependent kinase inhibitor 2A [CDKN2A]),^12^, ^13^, ^14^ as well as which mutations were highly prevalent within this cohort (eg, titin [TTN], FAT atypical cadherin 1 [FAT1]). We hypothesize that mutant TP53 will be associated with a more aggressive phenotype when compared to wild‐type (WT) TP53 and other gene mutations. Herein, this study investigates the correlation between several gene mutations in OCSCC with several pathologic features of malignancy (eg, ENE, PNI), to assess the prognostic implications of these mutations, and to assess potential utility in treatment stratification.

## Materials and Methods

### Search Strategy

Patient data were acquired from the cBioPortal for Cancer Genomics database. The following studies were queried for patient data: Broad, Science 2011,^15^ MD Anderson, Cancer Discovery 2013,^16^ The Cancer Genome Atlas (TCGA) Firehose Legacy,^17^ and Johns Hopkins, Science 2011.^18^ These were specifically selected to prevent overlapping data across studies, to ensure sufficient data were available for analysis, and to maintain consistency in the reference assembly used to sequence the human genome across all studies (Genome Reference Consortium Human Build 37) (GRCh37). Gene names and symbols are consistent with those provided by the United States National Center for Biotechnology Information.^19^

### Inclusion and Exclusion Criteria

Patients aged ≥18 years were included. Only studies involving HNSCC were queried. Included patients had primary tumor involvement of the oral cavity and/or its subsites, which included the alveolar ridge, buccal mucosa, floor of mouth, hard palate, lip, and oral tongue. Patients with missing data for the primary tumor site were excluded. All data were anonymized. This study met criteria for nonhuman subject research per the protocol of the Institutional Review Board of Rutgers New Jersey Medical School, Newark, New Jersey, and therefore, was exempt.

### Data Analysis

Data from the included studies were collected from cBioPortal and imported into IBM SPSS v29. An ideal sample size of 385 was calculated using the Qualtrics sample size calculator.^20^ Univariate analyses were performed using chi‐square, Fisher's exact test, and two‐tailed independent samples *t* tests, where appropriate, to assess for correlations between age, sex, race, smoking status, PNI, ENE, primary site, tumor stage, and tumor mutational burden (TMB) with each gene mutation type. TMB was grouped into TMB low (<10 mutations/megabase) and TMB high (≥10 mutations/megabase) based on the KEYNOTE‐158 trial definition.^21^ Metastatic spread was not included in the univariate analysis due to insufficient sample size. Survival curves amongst individual gene mutations were estimated using the Kaplan‐Meier (KM) method^22^ and were further assessed using Cox regression analysis. Patients with M1 or MX staging were excluded from survival analyses. Survival analysis for Casp8 mutations was unable to be assessed due to insufficient data. Criteria for disruptive and non‐disruptive TP53 mutations were based on the definitions provided by Poeta et al,^12^ apart from including both conservative and non‐conservative missense mutations. Logistic regression analyses were then conducted to assess for correlation between gene mutation presence, ENE, and PNI while controlling for age, sex, race, and smoking status.

## Results

### Overall Patient Characteristics

In total, 423 patients were included in this study. Mean diagnosis age was 61.5 years (range 19.0‐90.0), with 66.9% being male and 89.3% being white. In total, 74.1% were former or current smokers, and the most commonly involved primary site was the oral tongue (38.1%). Amongst those with data available, the most common AJCC tumor stages in the cohort were T4 (32.9%), N2 (26.7%), and M0 (77.5%), making stage 4 the most common overall stage (52.5%). There were 2 patients with distant metastasis and 12 with unmeasured metastasis. The most common gene mutation was TP53 (72.3%), which accounts for disruptive TP53 mutations (43.0%) and non‐disruptive TP53 mutations (29.3%), followed by TTN (31.2%) and FAT1 (22.0%). The median TMB was 3.03, and the TMB low group (<10 mutations/megabase) comprised 94.6% of the cohort. Positive ENE was seen in 27.5% of patients and positive PNI in 55.3% (Table 1).

**Table 1:** Demographic and Clinical Data of Overall Cohort

	Total
Subjects, N	423
Age in years, mean (range)^a^	61.5 (19.0‐90.0)
Sex, n (%)^a^	
Male	241 (66.9)
Female	119 (33.1)
Race, n (%)^a^	
White	276 (89.3)
Black	22 (7.1)
Other^b^	11 (3.6)
Smoking status, n (%)^a^	
Non‐smoker	107 (25.9)
Smoker	307 (74.1)
Overall survival status, n (%)	
Living	190 (52.8)
Deceased	170 (47.2)
Perineural invasion (PNI), n (%)^a^	
Negative	130 (44.7)
Positive	161 (55.3)
Extranodal extension (ENE), n (%)^a^	
Negative	203 (72.5)
Positive	77 (27.5)
Tumor mutational burden (TMB), median (range)	3.03 (0.07‐106.03)
TMB groups, n (%)	
TMB low (<10)	400 (94.6)
TMB high (≥10)	23 (5.4)
T stage, n (%)	
T1	20 (4.7)
T2	117 (27.7)
T3	101 (23.9)
T4	139 (32.9)
N stage, n (%)	
N1	72 (17.0)
N2	113 (26.7)
N3	2 (0.5)
M stage, n (%)	
M0	328 (77.5)
M1	2 (0.5)
MX	12 (2.8)
Tumor stage, n (%)	
Stage 1	12 (3.2)
Stage 2	84 (22.3)
Stage 3	83 (22.0)
Stage 4	198 (52.5)
Gene mutation, n (%)^c^	
Any TP53	306 (72.3)
Disruptive TP53	182 (43.0)
Non‐disruptive TP53	124 (29.3)
CDKN2A	88 (20.8)
Casp8	58 (13.7)
NOTCH1	75 (17.7)
TTN	132 (31.2)
FAT1	93 (22.0)
Primary site, n (%)	
Alveolar ridge	20 (4.7)
Buccal mucosa	25 (5.9)
Floor of mouth	71 (16.8)
Hard palate	7 (1.7)
Lip	3 (0.7)
Oral cavity	136 (32.1)
Oral tongue	161 (38.1)

Abbreviations: Casp8, caspase 8; CDKN2A, cyclin‐dependent kinase inhibitor 2A; FAT1, FAT atypical cadherin 1; NOTCH1, notch receptor 1; TP53, tumor protein p53; TTN, titin; WT, wild‐type.

^a^ Missing data, percentages are based on available data for each category rather than the total cohort.

^b^ Asian, American Indian, Alaska Native.

^c^ Sum of gene mutations is >100% because each sample may contain multiple mutations. This redundancy is also seen amongst TP53 mutation subtypes.

### Tumor Characteristics and Clinical Outcomes

On univariate analyses, patients with mutated TP53 appeared younger, bordering on statistical significance (60.7 vs 63.5 years, *P* = .069). Amongst TP53 patients, mutant TP53 was more often associated with adverse pathologic features; this included PNI (59.4% vs 44.3%, *P* = .021) and presence of ENE (31.0% vs 18.8%, *P* = .038). Similar relationships were seen when analyzing disruptive TP53 versus non‐disruptive TP53 versus WT TP53: presence of ENE was most frequent in disruptive TP53 (35.6% disruptive vs 24.4% non‐disruptive vs 18.8% WT, *P* = .025). PNI was more common in disruptive and non‐disruptive TP53 mutations compared to WT, although this was not statistically significant (52.4% disruptive vs 52.1% non‐disruptive vs 39.8% WT, *P* = .133) (Table 2).

**Table 2:** Demographic and Clinical Data by Tumor Protein p53 (TP53) Mutation

		WT TP53 (n = 117)	Any TP53 mutation (n = 306)	*P*‐value	WT TP53 (n = 117)	Non‐disruptive TP53 mutation (n = 124)	Disruptive TP53 mutation (n = 182)	*P*‐value
Age^a^	Age, years mean, [SE]	63.5 [1.2]	60.7 [0.8]	.069	62.6 [0.9]	61.7 [13.0]	60.0 [13.8]	.117
Sex, n (%)^a^	Male	63 (61.8)	178 (69.0)	.189	63 (61.8)	67 (63.2)	111 (73.0)	.108
	Female	39 (38.2)	80 (31.0)		39 (38.2)	39 (36.8)	41 (27.0)	
Race, n (%)^a^	White	73 (91.3)	203 (88.6)	.692	73 (91.3)	87 (92.6)	116 (95.9)	.164
	Black	4 (5.0)	18 (7.9)		4 (5.0)	7 (7.4)	11 (8.1)	
	Other^b^	3 (3.7)	8 (3.5)		3 (3.7)	0	8 (5.9)	
Smoking status, n (%)^a^	Non‐smoker	25 (21.9)	82 (27.3)	.262	23 (21.9)	30 (27.3)	50 (31.1)	.262
	Smoker	89 (78.1)	218 (72.7)		82 (78.1)	80 (72.7)	111 (68.9)	
Perineural invasion (PNI), n (%)^a^	Negative	44 (55.7)	86 (40.6)	.021	53 (60.2)	45 (47.9)	70 (47.6)	.133
	Positive	35 (44.3)	126 (59.4)		35 (39.8)	49 (52.1)	77 (52.4)	
Extranodal extension (ENE),n (%)^a^	No	65 (81.2)	138 (69.0)	.038	65 (81.2)	62 (75.6)	76 (64.4)	.025
	Yes	15 (18.8)	62 (31.0)		15 (18.8)	20 (24.4)	42 (35.6)	
Tumor stage, n (%)^a^	Stage 1	5 (4.7)	7 (2.6)	.535	5 (4.7)	5 (4.4)	2 (1.3)	.647
	Stage 2	22 (20.8)	62 (22.9)		22 (20.8)	24 (21.3)	38 (24.1)	
	Stage 3	20 (18.9)	63 (23.2)		20 (18.9)	25 (22.1)	36 (22.7)	
	Stage 4	59 (55.6)	139 (51.3)		59 (55.6)	59 (52.2)	82 (51.9)	
Tumor mutational burden (TMB), n (%)	TMB low	110 (94.0)	290 (94.8)	.760	110 (94.0)	120 (96.8)	170 (93.4)	.423
	TMB high	7 (6.0)	16 (5.2)		7 (6.0)	4 (3.2)	12 (6.6)	

Abbreviation: WT, wild‐type.

^a^ Missing data, percentages are based on available data for each category rather than the total cohort.

^b^ Asian, American Indian, Alaska Native.

Patients with mutant TTN were older in age compared to WT TTN (63.8 vs 60.3 years, *P* = .018). Mutant FAT1 patients were also older on average compared to WT FAT1 (66.6 vs 59.8 years, *P* < .001). High TMB was more commonly seen in mutant TTN patients compared to WT TTN (13.6% vs 1.7%, *P* < .001), as well as in mutant FAT1 compared to WT FAT1 (10.8% vs 3.9%, *P* = .010). Notably, 18/23 (78.3%) patients with high TMB in this cohort were found to have a TTN mutation (Table 3).

**Table 3:** Demographic and Clinical Data by Titin (TTN) and FAT Atypical Cadherin 1 (FAT1) Mutation

		WT TTN (n = 291)	Mutated TTN (n = 132)	*P*‐value	WT FAT1 (n = 330)	Mutated FAT1 (n = 93)	*P*‐value
Age^a^	Age, years mean, [SE]	60.3 [0.9]	63.8 [1.2]	**.018**	59.8 [0.8]	66.6 [12.2]	<**.001**
Sex, n (%)^a^	Male	160 (67.2)	81 (66.4)	.874	189 (69.5)	52 (59.1)	.072
	Female	78 (32.8)	41 (33.6)		83 (30.5)	36 (40.9)	
Race, n (%)^a^	White	175 (89.3)	101 (89.3)	.745	202 (89.0)	74 (90.2)	.915
	Black	13 (6.6)	9 (8.0)		17 (7.5)	5 (6.1)	
	Other^b^	8 (4.1)	3 (2.7)		8 (3.5)	3 (3.7)	
Smoking status, n (%)^a^	Non‐smoker	76 (26.8)	31 (23.8)	.530	85 (26.3)	22 (24.2)	.680
	Smoker	208 (73.2)	99 (76.2)		238 (73.7)	69 (75.8)	
Perineural invasion (PNI), n (%)^a^	Negative	81 (43.1)	49 (47.6)	.462	102 (45.3)	28 (42.4)	.676
	Positive	107 (56.9)	54 (52.4)		123 (54.7)	38 (57.6)	
Extranodal extension (ENE), n (%)^a^	No	139 (73.2)	64 (71.1)	.720	148 (69.8)	55 (80.9)	.075
	Yes	51 (26.8)	26 (28.9)		64 (30.2)	13 (19.1)	
Tumor stage, n (%)^a^	Stage 1	9 (3.5)	3 (2.5)	.111	10 (3.4)	2 (2.4)	.954
	Stage 2	58 (22.8)	26 (21.5)		64 (21.9)	20 (23.5)	
	Stage 3	65 (25.4)	19 (15.7)		64 (21.9)	19 (22.4)	
	Stage 4	124 (48.3)	73 (60.3)		154 (52.8)	44 (51.7)	
Tumor mutational burden (TMB), n (%)	TMB low	286 (98.3)	114 (86.4)	<**.001**	317 (96.1)	83 (89.2)	**.010**
	TMB high	5 (1.7)	18 (13.6)		13 (3.9)	10 (10.8)	

Abbreviation: WT, wild‐type.

^a^ Missing data, percentages are based on available data for each category rather than the total cohort.

^b^ Asian, American Indian, Alaska Native.

Patients with mutant NOTCH1 were older compared to WT NOTCH1 (64.8 vs 61.0 years, *P* = .042). Mutant NOTCH1 patients bordered on statistical significance for having high TMB compared to WT NOTCH1 (12.3% vs 5.5%, *P* = .052). Mutant CDKN2A patients were less likely to be current/former smokers compared to WT CDKN2A (62.0% vs 74.6%, *P* = .033) (Table 4). Patients with Casp8 mutation were older (69.8 vs 60.1, *P* < .001) and more likely to be female (51.9% vs 29.9%, *P* = .002) compared to WT Casp8. Mutant Casp8 patients were more likely to have high TMB compared to WT Casp8 (17.2% vs 3.6%, *P* < .001) (Table 5).

**Table 4:** Demographic and Clinical Data by Notch Receptor 1 (NOTCH1) and Cyclin‐Dependent Kinase Inhibitor 2A (CDKN2A) Mutation

		WT NOTCH1 (n = 348)	Mutated NOTCH1 (n = 75)	*P*‐value	WT CDKN2A (n = 335)	Mutated CDKN2A (n = 88)	*P*‐value
Age^a^	Age, years mean, [SE]	61.0 [0.8]	64.8 [1.8]	**.042**	62.2 [12.8]	60.5 [14.1]	.322
Sex, n (%)^a^	Male	175 (68.6)	38 (58.5)	.121	160 (66.9)	53 (65.4)	.803
	Female	80 (31.4)	27 (41.5)		79 (33.1)	28 (34.6)	
Race, n (%)^a^	White	219 (88.7)	57 (91.9)	.627	203 (88.3)	73 (92.4)	.589
	Black	18 (7.3)	4 (6.5)		18 (7.8)	4 (5.1)	
	Other^b^	10 (4.0)	1 (1.6)		9 (3.9)	2 (2.5)	
Smoking status, n (%)^a^	Non‐smoker	75 (30.2)	14 (22.2)	.209	59 (25.4)	30 (38.0)	**.033**
	Smoker	173 (69.8)	49 (77.8)		173 (74.6)	49 (62.0)	
Perineural invasion (PNI), n (%)^a^	Negative	107 (45.7)	23 (40.4)	.464	108 (47.0)	22 (36.1)	.128
	Positive	127 (54.3)	34 (59.6)		122 (53.0)	39 (63.9)	
Extranodal extension (ENE), n (%)^a^	No	134 (70.5)	39 (78.0)	.295	133 (72.3)	40 (71.4)	.901
	Yes	56 (29.5)	11 (22.0)		51 (27.7)	16 (28.6)	
Tumor stage, n (%)^a^	Stage 1	10 (4.0)	2 (3.1)	.874	11 (4.7)	1 (1.3)	.073
	Stage 2	62 (25.1)	15 (23.1)		55 (23.6)	22 (27.8)	
	Stage 3	50 (20.3)	16 (24.6)		43 (18.5)	23 (29.1)	
	Stage 4	125 (50.6)	32 (49.2)		124 (53.2)	33 (41.8)	
Tumor mutational burden (TMB), n (%)^a^	TMB low	241 (94.5)	57 (87.7)	.052	220 (92.1)	78 (96.3)	.192
	TMB high	14 (5.5)	8 (12.3)		19 (7.9)	3 (3.7)	

Abbreviation: WT, wild‐type.

^a^ Missing data, percentages are based on available data for each category rather than the total cohort.

^b^ Asian, American Indian, Alaska Native.

**Table 5:** Demographic and Clinical Data by Caspase 8 (Casp8) Mutation

		WT Casp8 (n = 365)	Mutated Casp8 (n = 58)	*P*‐value
Age^a^	Age, years mean, [SE]	60.1 [12.6]	69.8 [13.5]	<**.001**
Sex, n (%)^a^	Male	216 (70.1)	25 (48.1)	**.002**
	Female	92 (29.9)	27 (51.9)	
Race, n (%)^a^	White	231 (88.5)	45 (93.7)	.557
	Black	20 (7.7)	2 (4.2)	
	Other^b^	10 (3.8)	1 (2.1)	
Smoking status, n (%)^a^	Non‐smoker	90 (25.2)	20 (35.1)	.117
	Smoker	267 (74.8)	37 (64.9)	
Perineural invasion (PNI), n (%)^a^	Negative	109 (43.8)	21 (50.0)	.453
	Positive	140 (56.2)	21 (50.0)	
Extranodal extension (ENE), n (%)^a^	No	169 (71.0)	34 (81.0)	.183
	Yes	69 (29.0)	8 (19.0)	
Tumor stage, n (%)^a^	Stage 1	11 (3.4)	1 (1.9)	.808
	Stage 2	71 (21.9)	13 (25.0)	
	Stage 3	70 (21.5)	13 (25.0)	
	Stage 4	173 (53.2)	25 (48.1)	
Tumor mutational burden (TMB), n (%)	TMB low	352 (96.4)	48 (82.8)	<**.001**
	TMB high	13 (3.6)	10 (17.2)	

Abbreviation: WT, wild‐type.

^a^ Missing data, percentages are based on available data for each category rather than the total cohort.

^b^ Asian, American Indian, Alaska Native.

On multivariable analysis controlling for age, sex, race, and smoking status, mutant TP53 was associated with greater odds for positive ENE (odds ratio [OR] 2.61, 95% CI 1.05‐6.52, *P* = .039) and presence of PNI (OR 2.14, 95% CI 1.04‐4.42, *P* = .039). Disruptive TP53 patients also had greater odds of ENE compared to WT TP53 (OR 2.33, 95% CI 1.20‐4.51, *P* = .012) (Table 6).

**Table 6:** Logistic Regression: Pathologic Features of Malignancy Based on Gene Mutation

	Predictor^a^	Adjusted odds ratio	95% CI	*P*‐value
Any TP53 mutation	Extranodal extension (ENE)	2.61	1.05‐6.52	**.039**
	Perineural invasion (PNI)	2.14	1.04‐4.42	**.039**
	Age	0.98	0.95‐1.01	.119
	Sex	1.54	0.69‐3.48	.296
	Race	0.74	0.34‐1.59	.434
	Smoking status	0.41	0.17‐1.03	.059
Disruptive TP53 mutation^b^	Extranodal extension (ENE)	2.33	1.20‐4.51	**.012**
	Perineural invasion (PNI)	1.24	0.67‐2.29	.492
	Age	0.99	0.96‐1.01	.259
	Sex	1.25	0.63‐2.51	.526
	Race	1.13	0.58‐2.20	.730
	Smoking status	0.53	0.26‐1.07	.078

Abbreviation: TP53, tumor protein p53.

^a^ Reference: wild‐type gene.

^b^ Reference: wild‐type gene (excluding non‐disruptive TP53).

### Survival

**Figure 1:**
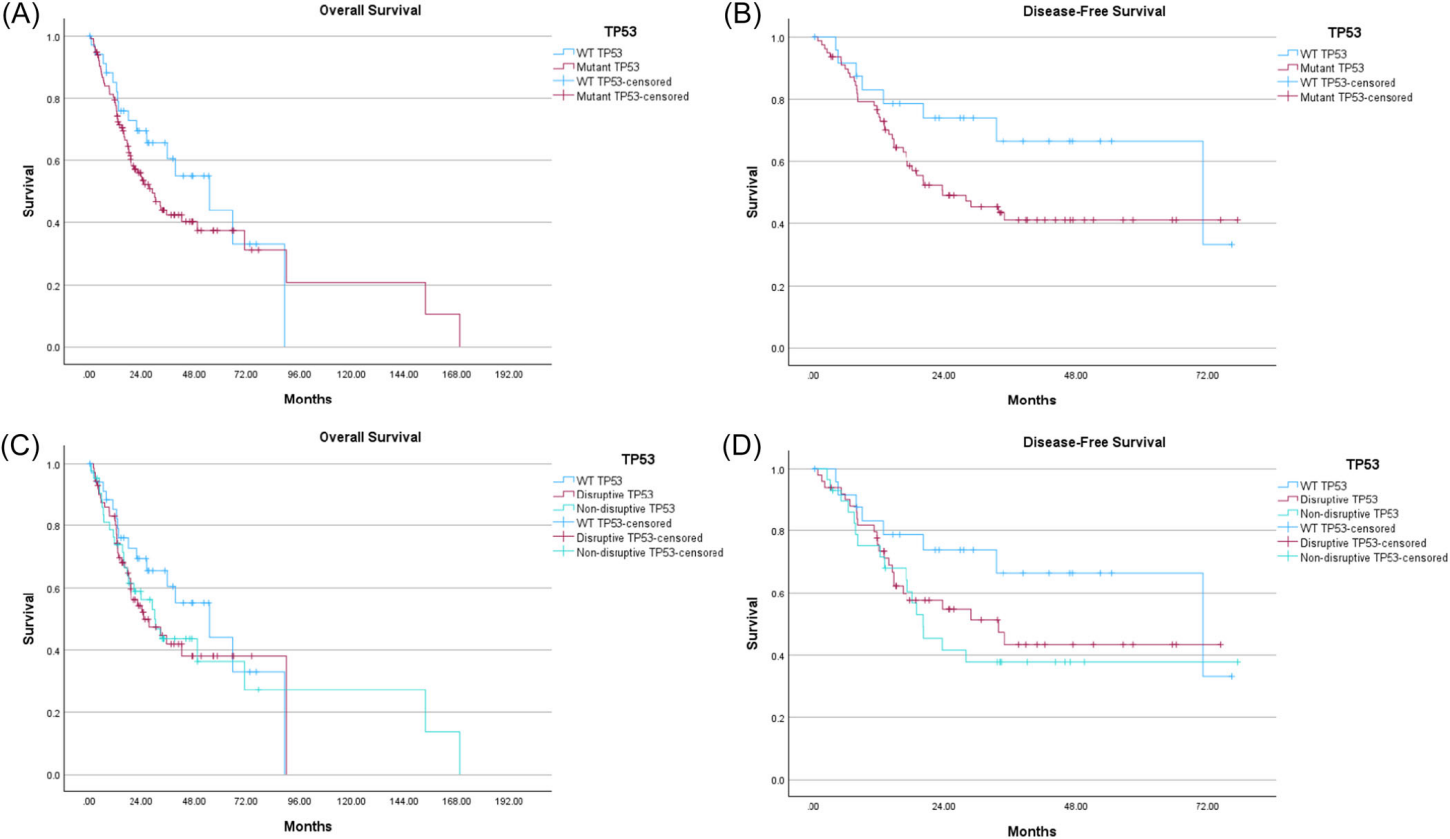
Kaplan‐Meier survival analyses: tumor protein p53 (TP53) mutations in stage 4 M0 oral cavity squamous cell carcinoma (OCSCC). WT, wild‐type.

On KM survival analysis amongst AJCC stage 4 patients without distant metastasis, median OS was shorter, but not statistically significant, in any TP53 mutation versus WT TP53 (29.0 vs 54.9 months, *P* = .296) and in disruptive TP53 versus non‐disruptive TP53 versus WT TP53 (25.1 vs 30.1 vs 54.9 months, respectively; *P* = .562) (Figure 1). Univariate Cox regression analyses of median OS were not significant for any TP53 (hazard ratio [HR] 1.34, *P* = .298), disruptive TP53 (HR 1.38, *P* = .289), nor non‐disruptive TP53 (HR 1.29, *P* = .430) when using WT TP53 as a reference. Mutant TTN, FAT1, NOTCH1, Casp8, and CDKN2A all demonstrated similar median OS compared to their respective WT genes (Table 7). Multivariable Cox regression analysis amongst stage 4 patients controlling for age, sex, and race did not reveal worse median OS for any TP53 mutation (HR 1.29, *P* = .373), disruptive TP53 (HR 1.36, *P* = .318), non‐disruptive TP53 (HR 1.20, *P* = .581), nor mutant CDKN2A (HR 1.29, *P* = .364) compared to their respective WT genes (Table 8).

**Table 7:** Univariate Cox Regression Analyses: Overall Survival by Mutation Type in Stage 4 M0 Patients

Predictor	Hazard ratio	95% CI	*P*‐value
Any TP53			
Wild type	REF	REF	
Mutant	1.34	0.77‐2.33	.298
TP53 detailed			
Wild type	REF	REF	
Non‐disruptive	1.29	0.68‐2.44	.430
Disruptive	1.38	0.76‐2.48	.289
TTN			
Wild type	REF	REF	
Mutant	1.08	0.69‐1.69	.729
FAT1			
Wild type	REF	REF	
Mutant	0.97	0.58‐1.64	.912
NOTCH1			
Wild type	REF	REF	
Mutant	1.20	0.69‐2.09	.518
CDKN2A			
Wild type	REF	REF	
Mutant	1.26	0.74‐2.15	.389

Abbreviations: CDKN2A, cyclin‐dependent kinase inhibitor 2A; FAT1, FAT atypical cadherin 1; NOTCH1, notch receptor 1; TP53, tumor protein p53; TTN, titin.

**Table 8:** Multivariable Cox Regression Analyses: Overall Survival by Mutation Type in Stage 4 M0 Patients

Predictor	Hazard ratio	95% CI	*P*‐value
Any TP53			
Wild type	REF	REF	
Mutant	1.29	0.74‐2.27	.373
Age	1.01	0.98‐1.03	.577
Sex			
Male	REF	REF	
Female	1.24	0.73‐2.09	.428
Race			
White	REF	REF	
Black	1.63	0.72‐3.67	.241
Other	0.71	0.17‐2.97	.644
TP53 detailed			
Wild type	REF	REF	
Non‐disruptive	1.20	0.63‐2.30	.581
Disruptive	1.36	0.74‐2.49	.318
Age	1.01	0.98‐1.03	.560
Sex			
Male	REF	REF	
Female	1.27	0.74‐2.16	.386
Race			
White	REF	REF	
Black	1.65	0.73‐3.74	.228
Other	0.70	0.17‐2.91	.622

Abbreviations: TP53, tumor protein p53; WT, wild‐type.

## Discussion

Understanding negative prognostic factors can help select patients for treatment intensification and can help in counseling. Gene mutations could play a role in predicting patient prognosis. Currently, the standard of care for stage III/IV resectable HNSCC is postoperative radiation therapy (PORT), with or without concurrent chemotherapy, and this is shifting to add both neoadjuvant and adjuvant immunotherapy in locally advanced HNSCC demonstrating a CPS ≥ 1.^7^, ^23^, ^24^, ^25^ A meta‐analysis of two randomized trials demonstrated that HNSCC patients who are most likely to benefit from PORT with concurrent chemotherapy are those with positive resection margins and/or ENE in regional lymph nodes.^25^ Even without adverse features of ENE or positive margins, many patients still have poor outcomes, and thus some researchers are exploring the potential benefit of adding chemotherapy for advanced‐stage OCSCC patients with the use of molecular testing for prognostic assessment and treatment stratification. Poeta et al demonstrated mutant TP53 to be associated with a decreased median 5‐year OS compared to WT TP53, independent of tumor stage (HR 1.7, *P* = .009).^12^ An ongoing clinical trial, EA3132, is investigating this topic by randomizing resected HNSCC without ENE or positive margins to radiation therapy versus chemoradiation therapy, and stratifying analysis based on TP53 mutation type (disruptive, non‐disruptive, and WT).^26^ Notably, Poeta et al did not report on the prevalence of adverse pathologic features (eg, ENE, PNI).

The incidence of the gene mutations analyzed in this study has been previously reported in a similar frequency as observed in our cohort. We found a mutation prevalence of 72.3% for TP53, 31.2% for TTN, 22.0% for FAT1, 20.8% for CDKN2A, 17.7% for NOTCH1, and 13.7% for Casp8. These frequencies are similar compared to recent studies using TCGA data, reporting an incidence of 66% for TP53, 35% for TTN, 21% for FAT1, 20% for CDKN2A, 16% for NOTCH1, and 10.9% for Casp8.^13^

Research has consistently shown that TP53 mutations, particularly those that disrupt the coding region of the gene, are associated with more aggressive tumor behavior and earlier onset.^12^, ^27^ Despite this, the current study did not find that TP53 mutant patients had a worse 5‐year prognosis on analysis of OS or DFS. There is some early separation of survival curves on KM analyses of TP53 mutations around the 2 to 3 year mark for both OS and DFS (Figure 1). However, these curves converge again at the 5‐year follow‐up. Possible explanations for this could include long‐term treatment toxicity, additional malignancy, or other diseases. This data cohort had limited information about adjuvant treatment history, so we are unable to control for any potential differences between groups with respect to receipt of radiation or chemoradiation. We are interested in the results of the EA3132 trial, which will be able to more definitively determine if disruptive TP53 mutants have a worse prognosis than those with WT TP53, and if the addition of chemotherapy to adjuvant radiation results in improved outcomes in these patients.

Differences in OS in OCSCC patients have been reported across other gene mutations in addition to TP53. Mutations including HNRNPH1 and WDR81 mutations correlate with improved OS in SCC of the tongue.^13^, ^28^ In contrast, NOTCH1, FAT1, and CDKN2A have been associated with worse OS in some studies of OCSCC patients.^12^, ^29^, ^30^, ^31^ A recent study showed worse OS in HNSCC patients with mutant Casp8, but this difference in survival was not seen when looking specifically at a TCGA cohort of OCSCC patients.^13^ The results of our study did not show an association of OS amongst AJCC stage 4 patients with any gene mutation (Figure 1 and Supplemental Figure S1, available online). These analyses did, however, find an independent association of any TP53 mutation and disruptive TP53 mutation with pathologic features of malignancy such as ENE and PNI (Table 6). Further investigations into the genomic landscape of OCSCC, its link to adverse pathologic features, and potential links to patient outcomes are needed.

In recent years, the identification of a biomarker to assess the prognosis and treatment responsiveness of patients with OCSCC has become a topic of significant interest.^12^, ^32^, ^33^, ^34^, ^35^ TMB is one such biomarker that has received significant attention across many solid tumor types. Studies support the use of TMB as a predictive biomarker, as high TMB has been associated with a better response to immune checkpoint inhibitor therapy.^4^ This improved response ultimately led to the tissue‐agnostic approval of pembrolizumab use in unresectable or metastatic solid tumors with a TMB ≥ 10 mutations/megabase, following the results of the KEYNOTE‐158 clinical trial.^21^ Notably, this study only included one OCSCC patient. We found that high TMB is rare in OCSCC (5.4% in this cohort), which concurs with prior studies investigating TMB in HNSCC. One study with ~40% of their HNSCC cohort being composed of OCSCC found that 21% of the cohort had TMB ≥ 10.^36^ Another study with ~25% of their cohort being OCSCC demonstrated a median TMB of 5.0,^37^ which is slightly higher than the median TMB of 3.03 in our cohort. These findings suggest that OCSCC tends to have a low TMB.

The role gene mutations play in average TMB in HNSCC is unclear. For example, in one study, mutant TP53 correlated with higher average TMB,^38^ yet lacked correlation in another.^36^ Other gene mutations, including CDKN2A, TTN, and FAT1, have been associated with higher average TMB.^37^, ^39^, ^40^ Our study found no association between mutant TP53 and TMB, but mutant TTN, FAT1, and Casp8 demonstrated significant associations with high TMB compared to WT, and mutant NOTCH1 bordered on statistical significance for higher TMB (2, 3, 4, 5). These discrepancies highlight the need for further research to determine if gene mutations in specific pathways are actually driving differences in TMB, or if other factors play a more significant role.

ENE is an important clinical factor to consider in the postoperative treatment of HNSCC, as demonstrated by the treatment benefit of adding cisplatin to PORT in these patients.^25^ Prior studies investigating the relationship between individual gene mutations and ENE in OCSCC have found a positive correlation between high‐risk TP53 mutations and the presence of ENE.^41^, ^42^ On multivariable analysis, we found that any TP53 mutation is associated with the presence of ENE (OR 2.61, *P* = .039) as well as disruptive TP53 mutations (OR 2.33, *P* = .012) (Table 6). We found no correlation between mutant TTN, FAT1, NOTCH1, CDKN2A, or Casp8 with ENE (Supplemental Table S1, available online). The association of mutant TP53 with ENE that we identified in this study raises the question as to whether the worse prognosis of mutant TP53 patients found in other studies is related to this adverse feature.

Another characteristic that has been linked with a poor prognosis in OCSCC is PNI.^43^ Gene mutations have been studied to determine their capacity to predict PNI to circumvent several diagnostic challenges, including human error, and the oversimplification of reporting PNI to simply presence versus absence, which excludes relevant details such as extent of invasion and size of the involved nerve.^43^, ^44^ One example is neural cell adhesion molecule (N‐CAM) expression, which has been positively correlated with the presence of PNI.^45^ Therefore, we attempted to identify gene mutations associated with PNI in this cohort, finding positive correlations between PNI and any TP53 mutation (Table 6), and this relationship bordered on statistical significance for mutant CDKN2A compared to WT CDKN2A (Supplemental Table S1, available online).

Limitations of this study include the use of older data collected from studies published in 2011‐2016, which used the reference assembly GRCh37. This has been replaced by GRCh38; however, this newer version has not yet been widely adopted by laboratories, and the accuracy benefit in sequencing well‐characterized mutations, such as those included in this study, is likely marginal.^46^ Some patients were missing clinical data, which may impact the results of statistical analysis. The calculations of TMB remain unstandardized across studies and commercial platforms, which may cause discrepancies amongst studies.^47^, ^48^, ^49^ The KEYNOTE‐158 trial only included one patient with OCSCC with high TMB,^50^ and thus our cohort does not represent the majority of the patients who were enrolled in the trial. Due to a limitation in data collection, the disruptive TP53 mutation group includes both conservative and non‐conservative missense mutations in the L2‐L3 DBD of TP53, potentially underestimating the impact of disruptive TP53 mutations in our analyses. Treatment data were insufficiently available to be included in this study, which prevented our ability to control for treatment differences amongst the cohorts.

## Conclusion

TP53 mutation was very common in OCSCC (72.3%) and was associated with the presence of high‐risk pathologic features including ENE and PNI. However, mutant TP53 was not associated with worse OS. TMB is low in OCSCC, with a median value of 3.03 and only 5.4% with a TMB ≥ 10. Further research into the genomic landscape of OCSCC and its impact on patient prognosis and treatment outcomes is needed.

## Author Contributions

**Joseph Celidonio**, conceptualization, data curation, formal analysis, writing—original draft preparation (lead); **Sree Chinta**, visualization, writing—original draft preparation (supporting); **John Sebastian de Armas**, visualization, writing—original draft preparation (supporting); **Dylan Roden**, conceptualization, methodology, formal analysis; writing—review & editing.

## Disclosures

### Competing interests

The authors declare no conflicts of interest.

### Funding source

The authors did not receive any funding for this project.

## Supporting information

Supplemental Table S1. Pathologic features of malignancy based on gene mutation.

Supplemental Figure S1. Kaplan‐Meier survival analyses: titin (TTN), FAT atypical cadherin 1 (FAT1), notch receptor 1 (NOTCH1), cyclin‐dependent kinase inhibitor 2A (CDKN2A) mutations in stage 4 M0 oral cavity squamous cell carcinoma (OCSCC).
