# Supplementary material for: Genetic Landscape of Oral Cavity Squamous Cell Carcinoma
Source: OTO Open. 2026 Jan 21;10(1):e70194. doi: 10.1002/oto2.70194 (PMC12821888; doi:10.1002/oto2.70194)
Supplement: Supplementary file 1 — Supplemental Table S1. Pathologic features of malignancy based on gene mutation. [file OTO2-10-e70194-s001.docx]

|  | | **Predictor^a^** | **Adjusted Odds Ratio** | **95% CI** | **p-value** |
| --- | --- | --- | --- | --- | --- |
| Mutant TTN | **Extranodal Extension (ENE)** | 1.10 | 0.57 to 2.12 | 0.786 |  |
|  | **Perineural Invasion (PNI)** | 0.86 | 0.47 to 1.59 | 0.640 |  |
|  | **Age** | 1.02 | 0.99 to 1.04 | 0.170 |  |
|  | **Sex** | 0.97 | 0.49 to 1.94 | 0.940 |  |
|  | **Race** | 1.38 | 0.72 to 2.68 | 0.335 |  |
|  | **Smoking Status** | 0.85 | 0.42 to 1.71 | 0.642 |  |
| Mutant FAT1 | **Extranodal Extension (ENE)** | 0.70 | 0.31 to 1.54 | 0.371 |  |
|  | **Perineural Invasion (PNI)** | 1.40 | 0.68 to 2.88 | 0.357 |  |
|  | **Age** | 1.04 | 1.01 to 1.07 | **0.007** |  |
|  | **Sex** | 0.67 | 0.31 to 1.46 | 0.314 |  |
|  | **Race** | 0.97 | 0.42 to 2.25 | 0.936 |  |
|  | **Smoking Status** | 1.11 | 0.49 to 2.53 | 0.804 |  |
| Mutant NOTCH1 | **Extranodal Extension (ENE)** | 0.54 | 0.23 to 1.27 | 0.159 |  |
|  | **Perineural Invasion (PNI)** | 1.16 | 0.56 to 2.40 | 0.698 |  |
|  | **Age** | 1.02 | 0.99 to 1.05 | 0.323 |  |
|  | **Sex** | 0.74 | 0.33 to 1.67 | 0.470 |  |
|  | **Race** | 1.01 | 0.43 to 2.38 | 0.975 |  |
|  | **Smoking Status** | 1.98 | 0.80 to 4.92 | 0.143 |  |
| Mutant CDKN2A | **Extranodal Extension (ENE)** | 1.23 | 0.58 to 2.59 | 0.595 |  |
|  | **Perineural Invasion (PNI)** | 1.94 | 0.93 to 4.06 | 0.078 |  |
|  | **Age** | 1.01 | 0.98 to 1.03 | 0.611 |  |
|  | **Sex** | 1.08 | 0.49 to 2.38 | 0.850 |  |
|  | **Race** | 0.67 | 0.27 to 1.68 | 0.395 |  |
|  | **Smoking Status** | 0.41 | 0.19 to 0.90 | **0.026** |  |
| Mutant Casp8 | | **Extranodal Extension (ENE)** | 0.42 | 0.15 to 1.24 | 0.116 |
|  |  | **Perineural Invasion (PNI)** | 0.86 | 0.37 to 1.97 | 0.716 |
|  |  | **Age** | 1.04 | 1.01 to 1.08 | **0.019** |
|  |  | **Sex** | 0.53 | 0.22 to 1.29 | 0.159 |
|  |  | **Race** | 0.96 | 0.35 to 2.67 | 0.937 |
|  |  | **Smoking Status** | 0.81 | 0.32 to 2.03 | 0.647 |
| ^a^Reference: Wild-type gene | | | | | |
