# Supplementary figures and images for: Genetic Landscape of Oral Cavity Squamous Cell Carcinoma
Source: OTO Open. 2026 Jan 21;10(1):e70194. doi: 10.1002/oto2.70194 (PMC12821888; doi:10.1002/oto2.70194)

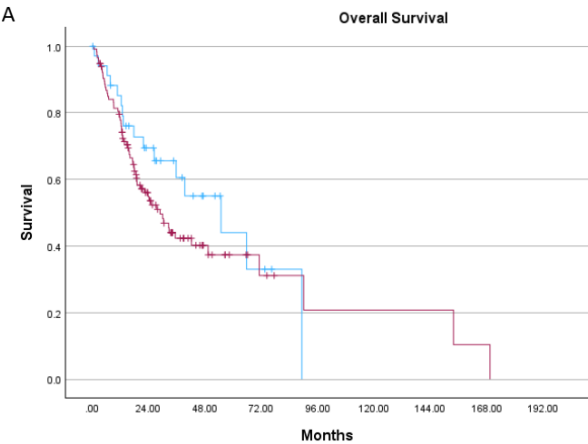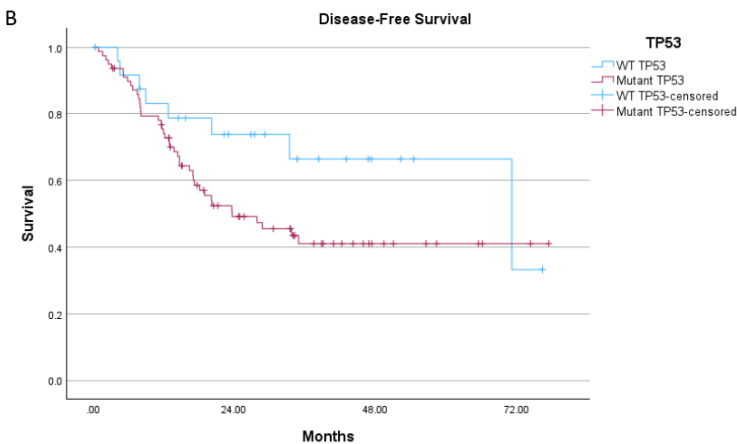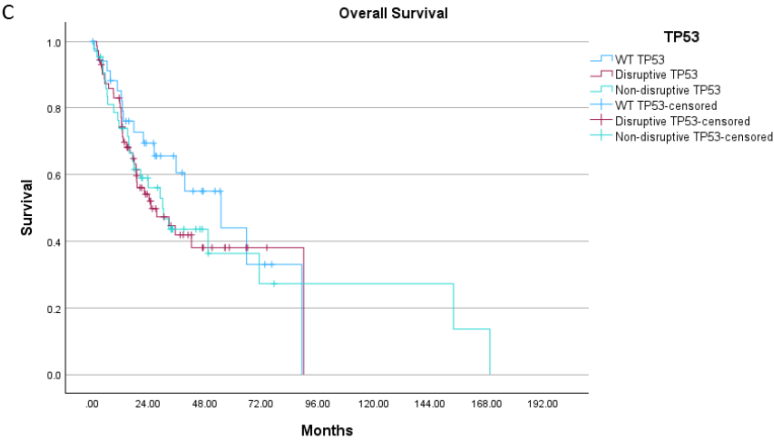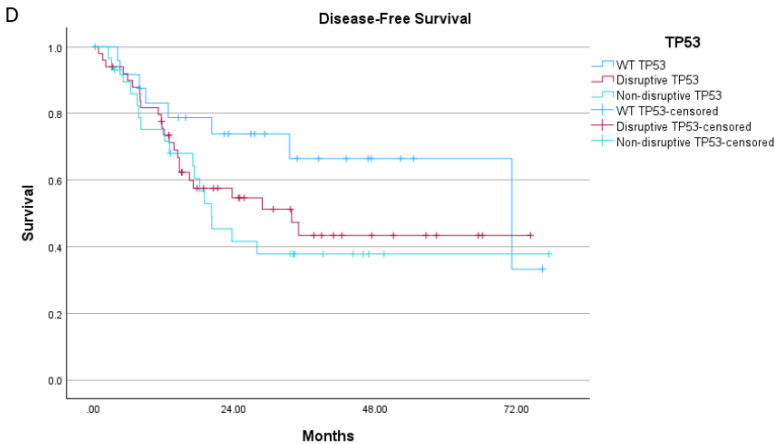

Supplement: Supplementary file 2 — Supplemental Figure S1. Kaplan‐Meier survival analyses: titin (TTN), FAT atypical cadherin 1 (FAT1), notch receptor 1 (NOTCH1), cyclin‐dependent kinase inhibitor 2A (CDKN2A) mutations in stage 4 M0 oral cavity squamous cell carcinoma (OCSCC). [file OTO2-10-e70194-s002.pdf]
